# The role of personal data value, culture and self-construal in online privacy behaviour

**DOI:** 10.1371/journal.pone.0253568

**Published:** 2021-07-16

**Authors:** Piers Fleming, Andrew P. Bayliss, S. Gareth Edwards, Charles R. Seger

**Affiliations:** School of Psychology, Centre for Behavioural and Economic Social Science, University of East Anglia, Norwich, United Kingdom; Heidelberg University, GERMANY

## Abstract

Personal data is ubiquitous in the digital world, can be highly valuable in aggregate, and can lead to unintended intrusions for the data creator. However, individuals’ expressions of concern about exposure of their personal information are generally not matched by their behavioural caution. One reason for this mismatch could be the varied and intangible value of personal data. We present three studies investigating the potential association between personal data value and privacy behaviour, assessing both individual and cross-cultural differences in personal data valuation, comparing collectivist and individualistic cultures. Study 1a, using a representative UK sample, found no relationship between personal data value and privacy behaviour. However, Study 1b found Indian (collectivist) participants’ privacy behaviour was sensitive to personal data value, unlike US (individualist) participants. Study 2 showed that in a UK sample, privacy behaviour was sensitive to personal data value but only for individuals who think of themselves as more similar to others (i.e., self-construe as similar, rather than different). We suggest those who prioritise group memberships are more sensitive to unintentional disclosure harm and therefore behave in accordance with personal data valuations—which informs the privacy concern-behaviour relationship. Our findings can suggest approaches to encourage privacy behaviours.

## Introduction

There is an increasing tension in the modern world between companies’ desire for our personal data and the importance of respecting individuals’ privacy. At time of writing the world’s most valuable companies in order are: Apple, Alphabet (including Google), Microsoft, Amazon and Facebook. Two of these companies are centred around personal data and it is a crucial part of the others’ businesses. For the individual, loss of control of personal data can lead to inadvertent harms such as junk email, identity theft or potentially damaging disclosures. It is unsurprising that we are averse to privacy intrusions into our physical space and social communications [1]. There are weak links, for example between need for privacy and Facebook use [2]; however, despite long-standing and cross-cultural evidence that most people report being ‘very’ or ‘extremely’ concerned about privacy, most people are incautious with their personal data [3]. Despite a range of studies on this topic there is not yet a consensus view on whether privacy concern meaningfully predicts privacy behaviour–the ‘privacy paradox’. Even meta-analytic reviews come to mixed conclusions. Some support the privacy paradox [4]; others find privacy concern does predict privacy management to some degree with positive associations between privacy concern and privacy literacy, and self-reported protective behaviour and/or intention; however, even then not all privacy behaviour is predicted by privacy concern, such as with social media [5]. These findings have encouraged a shift from the concept of privacy as a general threshold to a more nuanced context-specific privacy management view [6, 7].

Underlying evidence for the privacy paradox includes findings that most people will sell their personal data cheaply or in return for ‘free’ services such as use of a social network [8], for shopping discounts [9] or for free pizza [10]. A revealed preference approach would suggest that, despite claims to the contrary, people are not actually concerned about the privacy of their personal data. In fact, when privacy concern and behaviour is measured for the same people the relationship between stated concern and actual behaviour is surprisingly weak [11]. Either people are overstating their concern, or they are being more casual in their behaviour than their attitude suggests. The concept of ‘privacy calculus’ has been usefully employed and supported as an explanation for privacy concern and behaviour [12]. This account explains that disclosures are a rational outcome of weighing different aspects of privacy disclosure including the risks and benefits of sharing [13]. In this paper we focus on an aspect which may underlie individual judgments of risk and benefit and the consequent level of protective behaviour, our valuation of personal data.

Personal data value is distinct from privacy concern or privacy risk. We can be generally concerned that external organisations hold our personal data and that there is a high likelihood that they will share it without our permission. However, some of that personal data is precious to us, e.g. our bank account details or political beliefs while other personal data might be less valuable e.g. details of our Sunday lunch or one of our email addresses. Although we might expect higher personal data valuations to be associated with higher privacy concern or privacy risk that is not necessarily the case because that data may be more secure.

It is difficult to value personal data because it is intangible and generally gathered and used in a way which is opaque to the data creator. People have difficulty in evaluating the complex trade-offs around privacy protection, and personal data valuations are characterised as highly uncertain [14]. One reason for this uncertainty might be the difficulty of evaluation of data collected invisibly by computing devices in our environment and the intangibility of their cost. By contrast the value of the services offered in exchange for personal data, such as online search and social networks, are easier to appreciate. These conflicting feelings may lead to instability in actions and attitudes towards privacy, as well as increasing the irrationality of decision making [4]. However, there is research which attempts to measure people’s valuations of privacy and the consequences of those valuations in a concrete way.

Experimental studies into people’s privacy valuations reveal that they are varied, contextually influenced and typically low. When considered across a group of individuals, a typical pattern of valuations for concrete personal data includes a substantial group who will sell data at any price, and a substantial group who will not sell at any price, with a range of values in between [15, 16]. There is some overlap here with wider privacy attitudes which have divided people into privacy advocates, individualists and indifferents [17]. Generally, there are significant individual differences in any sort of valuation judgment [18], and these can be related to a variety of personality and cultural factors [19, 20]. We conclude that there are substantial individual differences in the valuation of personal data, with potential consequences for behaviour.

Valuations have been shown to be sensitive to the type of data in meaningful ways. Compared to other types of data, personal location/address [16, 21, 22]; and socially unacceptable behavioural data are highly valuable [23, 24]. In general, interactions are more valuable than shopping preferences [21, 25]. Thus, data that could cause us harm if disclosed is generally valued highly. We anticipate greater efforts to protect such valued data.

However, typical valuations of personal data are low, with many people willing to pay only a small premium to avoid disclosing information e.g. 1% extra, 1–2 euros or sometimes nothing at all [26, 27]. Furthermore, valuations have been shown to vary with question order or question context, which demonstrates considerable malleability in willingness-to-sell values [15, 28]. This implies that people are not robust in their valuations of their personal data.

One reason for the concern-behaviour mismatch, and inconsistent or low valuation of personal data might relate to the samples used. Recent research has highlighted the importance of individual socio-demographic differences in managing the complexity of social media disclosure and connection [6]. Existing research has been conducted primarily on western cultures which tend to have an overall individualistic outlook at a macro, cultural level [29]. Within that individualistic culture, in which individual achievement, rights and independence are prioritised, it may be that disclosures which affect interdependent relationships are less important and so privacy behaviour does not necessarily follow directly from concern [30]. Conversely, collectivist societies prioritise group achievement and interdependence, and so an individual within this culture may be more inclined to protect data which they value as it pertains to the cohesion of group dynamics.

There is wider pre-existing evidence of cross-cultural differences in the value of personal information and related behavioural regulation. Personal belief is a ready example of information that an individual can largely keep private, if desired. Greater control of behavioural expression in the form of conformity has been found amongst participants from collectivist countries [31]. People with lower levels of individualism been shown to demonstrate less trust in a social network, leading to a reduction in the desire to share information [32]. In collectivist cultures disclosing personal information or feelings outside one’s close circle may be less common than in individualistic societies. Sharing self-individuating information may reflect poorly on the individual’s group or their position in it [33]. Personal information, such as one’s emotional state, has greater value in collectivistic cultures; this greater value is associated with greater regulation and lower norms of interpersonal emotional expressions, particularly amongst ingroups [34]. We anticipate that this value and associated personal regulation will extend to personal data.

Currently, evidence regarding cross-cultural privacy concern is mixed, potentially in part due to a multitude of privacy-relevant factors which vary between countries (e.g. laws, wealth, and experience, as well as cultural values [35–37]). Despite considerable individual differences [38] evidence to date has typically measured individualism at a country-wide level as one aspect of Hofstede’s classic four-dimensional scale or one of two dimensions in a more recent 18-dimensional scale [29, 39]. Some studies suggest that privacy concern is associated with greater individualism [40, 41]. By contrast other studies suggest privacy concern is larger in groups with greater collectivism [35, 42–44]. Relatedly, there is some evidence that individualism is associated with reduced governmental privacy regulation [45]. Finally, there are some studies which find no difference in privacy concern between collectivist and individualist groups [46].

While the evidence for cross-cultural privacy *concern* is mixed, we believe the importance of privacy to inter-dependent relationships will lead to greater *value*, and that value and culture in combination will lead to more cautious behaviour around disclosure. There is some support for this view. One small cross-cultural study of value found that the most collectivist country valued personal data the highest [47]. Two large scale studies of *behaviour* have found that collectivism (as measured on a national level) is associated with: greater sensitivity to privacy settings for self-disclosure-behaviour on twitter [48], and greater use of privacy settings on Facebook [37]. One demonstration of this suggested mechanism is a cross-cultural study demonstrating that increased privacy intention from collectivist countries was largely moderated by social network connection importance–which equates to privacy value [49]. It is therefore intuitive to hypothesise a link between value and privacy behaviour whereby data that is highly valued is favourably protected, which may only bear out in collectivist samples.

Privacy concern, value and behaviour are complex. In one study, privacy behaviour relating to web-browsing behaviour was lower in Korean students (collectivist) compared to US students [46]. This pattern was also associated with an increased belief action disassociation for the Korean students. However, this would be consistent with our account if web-browsing behaviour is viewed as low value perhaps because they are not associated with personal beliefs or emotion. By contrast a study comparing Hispanic and non-Hispanic white populations political self-disclosure on social media found that greater self-disclosure amongst Hispanics (collectivist–see [50]) led to increased likelihood of blocking or unfriending with people opposing political attitudes, whereas the opposite was true of non-Hispanics [6]. This is consistent with potentially valuable personal belief information being associated with increased privacy behaviour to protect culture-based personal standing in one’s community.

A more fine-grained account of cultural differences can be obtained by considering individual self-construals [51]. Self-construal is your interpretation of what type of person you are including self-definition, relationship to others, and sources of self-esteem; the development of these is held to be culturally influenced, as with any individual difference, individuals within the same society can vary widely on these dimensions [52]. People have both independent and relationship-oriented self-construals but culture can nurture one over the other. Independent self-construals are characterised by self-definition in terms of stable internal traits that set the individual apart (e.g. creative). Relationship-oriented self-construals self-define by relationships of group memberships that the individual belongs to (e.g. son/Asian American). The differing motivations that come from the prioritisation of different self-construals cross-culturally imply greater adaptation of behaviour to belong in collectivist cultures and behaviour more consistent with self-beliefs in individualist cultures [53]. This means that collectivist cultural differences should be reflected in individual self-construal differences. We expect that relationship-oriented self-construals, which are more focussed on maintaining interpersonal relationships, will be more sensitive to privacy and self-presentational control [54].

This paper examines the role of personal data value as a predictor of privacy behaviour. We believe that personal data value might predict privacy behaviour in addition to privacy concern–and therefore partly explain the relationship between privacy concern and privacy behaviour. We further suggest that cultural or individual differences predict personal data valuation and their influence on privacy behaviour.

### Overview

Study 1a examines a general relationship between privacy behaviour and personal data value as well as privacy concern using a representative UK sample. Study 1b samples US and Indian crowd-sourced populations to evaluate cultural differences in the privacy value–behaviour association. The same measures are used as in Study 1a with the addition of country of residence, allowing for analysis of the interaction of personal data value and country. Finally, Study 2 samples a UK crowd-sourced population to predict privacy behaviour using privacy concern, personal data value, as well as individual differences in self-construal and the interaction of personal data value and self-construal. Self-construal was used as a possible explanation of US–Indian cultural differences in Study 1b.

## Study 1a –UK representative pilot

This study examined the baseline influence of personal data value and privacy concern on privacy behaviour as well as the inter-relations between personal data value and other personal data perceptions. Although personal data value is typically low, variable and contextually influenced it is still considered a meaningful psychological construct [4] and while distinct from privacy concern it should logically be associated with related concepts. This study tested the association between personal data value and perceptions of ownership, legal ownership, control and security. These perceptions were chosen because they are aspects of personal data that prima facie should be positively linked to value. People are unlikely to value their personal data if they believe that they don’t own it, or legally own it, or control it, or can keep it secure. By examining these values, we can determine the relative importance of these components and determine if there are meaningful differences between them.

Personal data value may also be associated with privacy behaviour. It may provide additional information that partly explains the pattern of evidence associated with the privacy paradox in which privacy concern is only weakly linked to privacy behaviour. This is the first investigation to consider personal data value in relation to the privacy concern and its association with prediction of behaviour. An individual is more likely to take action to protect their belongings (including data) if they are concerned about likely loss or damage or simply because they perceive their belongings as valuable [55]. However, it may be that personal data value uncertainty/variability diminishes this influence–particularly in an individualistic UK sample who have low initial privacy valuations. The personal data value, privacy concern, privacy behaviour link will be tested.

### Method

#### Participants

The UEA School of Psychology ethics committee approved these studies: 2016-0040-000316. Consent was obtained online at the commencement of the studies. A sample of UK residents were invited to take part in the study via a market research company. Quotas were used to recruit an approximately representative sample of the UK population across ages, gender and location. The target sample was 500 participants and recruitment was stopped shortly after the target was achieved. The final sample consisted of 525 participants; 942 began the survey, 363 participants were removed for failing attention filters, 42 withdrew/failed to complete the survey and 12 were removed for a total completion time under five minutes (under 1/3 median response time). Although a substantial percentage of participants failed the attention check this is not uncommon in psychological research [56]. The final sample was 50.9% female with an average age of 46.8 years (*SD* = 16.06). Median completion time was 15 minutes.

#### Materials and procedure

In all studies presented in this paper, participants completed online consent, followed by personal data perception questions about perceived ownership, perceived control, perceived legal ownership, perceived security and perceived value across 3 types of data (location data: “I feel like I own a week’s GPS coordinates of myself…”, a ‘selfie’: “I am in control of a selfie…”, an authored message: “I legally own a sent text message”) and across 6 types of situation–half were classified as proximal (temporally close: “I have sent during the last week”, spatially close: “…stored on a personal device (e.g. a running watch)”, private: “…on my phone”) and half were classified as distal (temporally distant: “…I have taken 3 years ago”, spatially distant: “…on cloud storage”, public: “…I have posted on Facebook”), See S7 for this question set. The original intention was to examine proximal and distal responses separately. However, in the first study the correlation between the two scales was high (*r* = .93, *p* < .001, n = 525) and in all studies the same pattern of results is found using the aggregated value scale or using either of the two sub-scales, therefore only the aggregated scales have been presented. Scale reliability scores are available in Table 1. These questions were presented in random order on a 7-point Likert-type scale from strongly disagree to strongly agree. Higher scores indicate greater data perceptions on that dimension (e.g. greater perceived value).

**Table 1:** Cronbach’s alpha, mean, standard deviation and correlation with value for the 5 personal data perceptions across UK (Studies 1a & 2) and USA and India (Study 1b).

Personal Data Perception (18–126)	UK—Study 1a				US				India				UK—Study 2			
	α	*M*	*SD*	*r*	α	*M*	*SD*	*r*	α	*M*	*SD*	*r*	α	*M*	*SD*	*r*
Personal Data Value	.96	69.15	24.55		.94	73.84	21.88		.88	95.39	13.79		.93	77.91	20.17	
Perceived Ownership	.95	73.20	22.48	.81	.93	76.51	20.53	.69	.88	93.31	14.59	.79	.93	81.60	19.58	.68
Perceived Legal Ownership	.95	72.19	22.43	.76	.94	75.11	20.83	.66	.90	92.22	15.80	.68	.94	80.10	21.01	.57
Perceived Control	.94	69.12	21.45	.68	.91	74.38	18.86	.54	.90	88.57	16.88	.54	.90	77.66	17.97	.58
Perceived Security	.95	67.60	21.23	.63	.94	69.91	20.22	.54	.91	85.66	17.52	.50	.92	73.47	18.71	.47

Note the reported correlation coefficients (column ‘r’) are the correlations between personal data value with the other four personal data perceptions by country; all are significant, *p* < .001.

Participants were subsequently asked to complete a 15-item concern for information privacy scale (CFIP) on a 7-point Likert-type scale from strongly disagree to strongly agree, [57], e.g. “I’m concerned that companies are collecting too much personal information about me” (α = .942). The CFIP is an established scale which has been used with UK participants, as we do in study 1a and 2 [58] but was originally validated with US samples, as we do in Study 1b [57] and has been used with Indian participants, as we do in Study 1b [59]. Participants also completed a self-reported privacy behaviour scale [60], including the original 12 items of the scale e.g. “Do you clear your browser history regularly?” plus an additional three items: “Do you encrypt your data (e.g. on laptop, phone)? Do you make an effort to ensure your social media privacy settings match your preferences? Do you have multiple social media accounts to compartmentalise your life (e.g. 2 twitter accounts, one ‘professional’, one ‘private’)?” All participants answered in English to reduce potential translation issues. All 15 items were scored on a 5-point scale from never to always, with good score for internal consistency (α = .855).

Between these two blocks of questions participants were asked to complete filler questions including two personality measures–one for social desirability (a 13-item scale, [61] and a ten-item personality inventory [62] as well as basic questions about attitudes to sharing information online, and a trust scale, totalling 14-items. The final questions asked about demographics and familiarity with technology.

Two attention filters were included during the study, about one third and two thirds of the way through, e.g. *Please select never for this statement*, participants were automatically ejected from the questionnaire if they answered incorrectly. The attention filter criteria were decided prior to the study.

### Results

As all three studies measured personal data perceptions across the five dimensions. we can compare them as shown in Table 1. Correlations revealed that perceived ownership, perceived legal ownership, perceived control and perceived security correlated highly with personal data value (see Table 1, *r* >.6, *p* < .001). Given the very high shared variance between the components it was not possible to include them separately in subsequent analyses with personal data value.

Initial correlations between the research variables revealed no association between privacy concern (CFIP) and personal data value (*r* (523) = .021, *p* = .625) nor any association between privacy behaviour and personal data value (*r* (523) = .031, *p* = .484) but a significant positive association between privacy concern (CFIP) and privacy behaviour (*r* (523) = .192, *p* < .001).

A multiple regression was carried out to predict self-reported privacy behaviours based on privacy concern (CFIP) and personal data value for the UK participants in this study (see Table 2). Unlike privacy concern, personal data value did not directly predict privacy behaviour for the representative UK sample. The same pattern of results is found if the available scores from attention-filtered participants were also included.

**Table 2:** Regression predicting privacy behaviour from demographic variables, privacy concern, and personal data value, Study 1a.

Variable	B	95% CI		SE	Beta	t	*p*	F	R-square
Constant	29.224	(21.736,	36.712)	3.812		7.667	< .001	6.059	.045***
Age	0.052	(-0.010,	0.114)	0.032	.074	1.635	.103		
Gender (1 = Female)	-0.141	(-2.071,	1.790)	0.983	-.006	-0.143	.886		
Privacy Concern (CFIP)	0.160	(0.085,	0.236)	0.038	.183	4.184	< .001		
Personal Data Value	0.021	(-0.019,	0.061)	0.020	.046	1.041	.298		

Note–Personal data value was mean centred.

*** *p* < .001. For all regressions reported in this paper, the pattern of results remains the same if technological familiarity is controlled for.

### Discussion

Privacy concern was associated with privacy behaviour and personal data value was not. This is in contrast to the predictions of the privacy paradox. As expected, personal data value was distinct from privacy concern. However, in this study personal data value did not contribute to improving the prediction of privacy behaviour. Overall, the R^2^ value of .045 for the model suggests considerable, unexplained variance. Personal data value was strongly related to our data perception measures (perceived ownership, perceived legal ownership, perceived control and perceived security). We can infer that insecure, uncontrolled and unowned personal data has less perceived value. Self-reported privacy behaviours from the privacy behaviour scale were predicted by the CFIP privacy concern scale but not by personal data value. There was no effect of personal data value on privacy behaviour.

We encourage future investigations to include further control or explanatory variables such as education, income, or uncertainty avoidance to more fully assess the unexplained variance apparent in this study. However, given that these variables are not the focus of theoretical interest in this study we have omitted them because they might vary across countries and the self-construals of interest–which are considered in the remaining studies. The introduction of these variables might, in fact, introduce bias or eliminate real cross-cultural differences [63], which is our interest in Study 1b.

## Study 1b –India, USA crowd-sourced sample

Study 1b investigates the relationship of personal data value with behaviour across cultures. Study 1a failed to find a significant association between personal data value and privacy behaviour with an individualistic UK sample. This may be attributed to a weak valuation of personal data in an individualistic culture resulting in a diminished role in decision-making. We will investigate this directly by examining populations with contrasting cultures. It is known that individualistic and collectivistic societies and values have different perspectives on group norms, communication and potentially personal privacy concern [6, 45, 46, 64, 65].

Individuals from collectivistic societies such as India are more likely to disclose personal information than individuals from individualistic societies such as America [66]. It seems likely that in a collective society in which group membership is more important personal data will also be more valued, because sharing personal information strengthens groups and builds mutual liking and understanding [67]. It is therefore likely that the greater personal value of data in a collectivist society will be more likely to predict privacy behaviour. Study 1b tests the perceived privacy concern, value, and behaviour relationship amongst US and Indian participants recruited from Amazon’s mechanical Turk.

### Method

#### Participants

This study was also approved by UEA School of Psychology ethics committee: 2016-0040-000316. Consent was obtained online at the commencement of the study. A sample of USA and Indian residents were invited to take part in the study via the Amazon mechanical Turk online platform. They were paid $2 each to participate. The final US sample consisted of 326 participants: 366 began the survey, 33 failed attention filters, 2 withdrew and 5 were removed for a total completion time under five minutes (as in Study 1a). The final Indian sample consisted of 305 participants: 411 began the survey, 63 were removed for failing attention filters, 39 withdrew and 4 were removed for a completion time under five minutes. All participants answered in English to reduce potential translation issues.

The US sample was 58.3% male, with an average age of 34.3 years (*SD* = 10.75). Median US participant completion time was 14.2 minutes. The Indian sample was 73.8% male with an average age of 32.2 years (*SD* = 9.57). Median Indian participant completion time was 20.4 minutes.

#### Materials and procedure

The procedure was identical to Study 1a, only country specific questions were altered to be appropriate (e.g. demographics). Survey scale reliabilities remained high for the CFIP (α_USA_ = .924, α_India_ = .911) and privacy behaviour scale (α_USA_ = .868, α_India_ = .866), see also Table 1.

### Results

As this study examines cross-cultural differences, US, UK and Indian personal data perceptions across the five dimensions were compared across studies 1a,1b and 2 using ANCOVAs. Indian participants rated data perceptions more highly than UK and US participants in every case, controlling for age and gender (*F* > 40, *p* < .001), see Table 1 for means. This supports one hypothesis–that Indian participants will value personal data more highly than UK or US participants. Furthermore, technological familiarity was significantly lower in the representative UK (Study 1a) sample (*M* = 44, *SD* = 16) compared to the US (*M* = 56, *SD* = 11) and Indian mTurk samples (*M* = 61, *SD* = 9); *F* = 192.5, *p* < .001.

Preliminary correlations revealed a different pattern to study 1a (see Table 3). Personal data value, privacy concern and privacy behaviour were all inter-related. Furthermore, the associations of personal data value with privacy concern (*z* = 2.13, *p* = .033) and with privacy behaviour (*z* = 2.81, *p* = .005) were stronger for participants in India while the privacy behaviour-privacy concern direct association was stronger for US participants (*z* = 3.06, *p* = .002).

**Table 3:** Correlation coefficients for research variables in Study 1b.

		Correlation coefficients _(Confidence Intervals)_					
Variables		US participants		India participants		All participants	
Personal Data Value	Privacy Concern (CFIP)	.195_(.088, 297)_	***	.352_(.250, .447)_	***	.202	***
Personal Data Value	Privacy Behaviour	.143_(.035, .248)_	***	.353_(.251, .448)_	***	.227	***
Privacy Behaviour	Privacy Concern (CFIP)	.406_(.311, .493)_	***	.184_(.073, .290)_	***	.300	***
Personal Data Value	Country^a^					.529	***
Privacy Concern (CFIP)	Country^a^					-.027	
Privacy Behaviour	Country^a^					.087	*

Note

* *p* < .05

*** *p* < .001.

^a^ These correlation coefficients use Spearman’s rho because country is categorised as 0 (US) or 1 (India)

**Fig 1:**
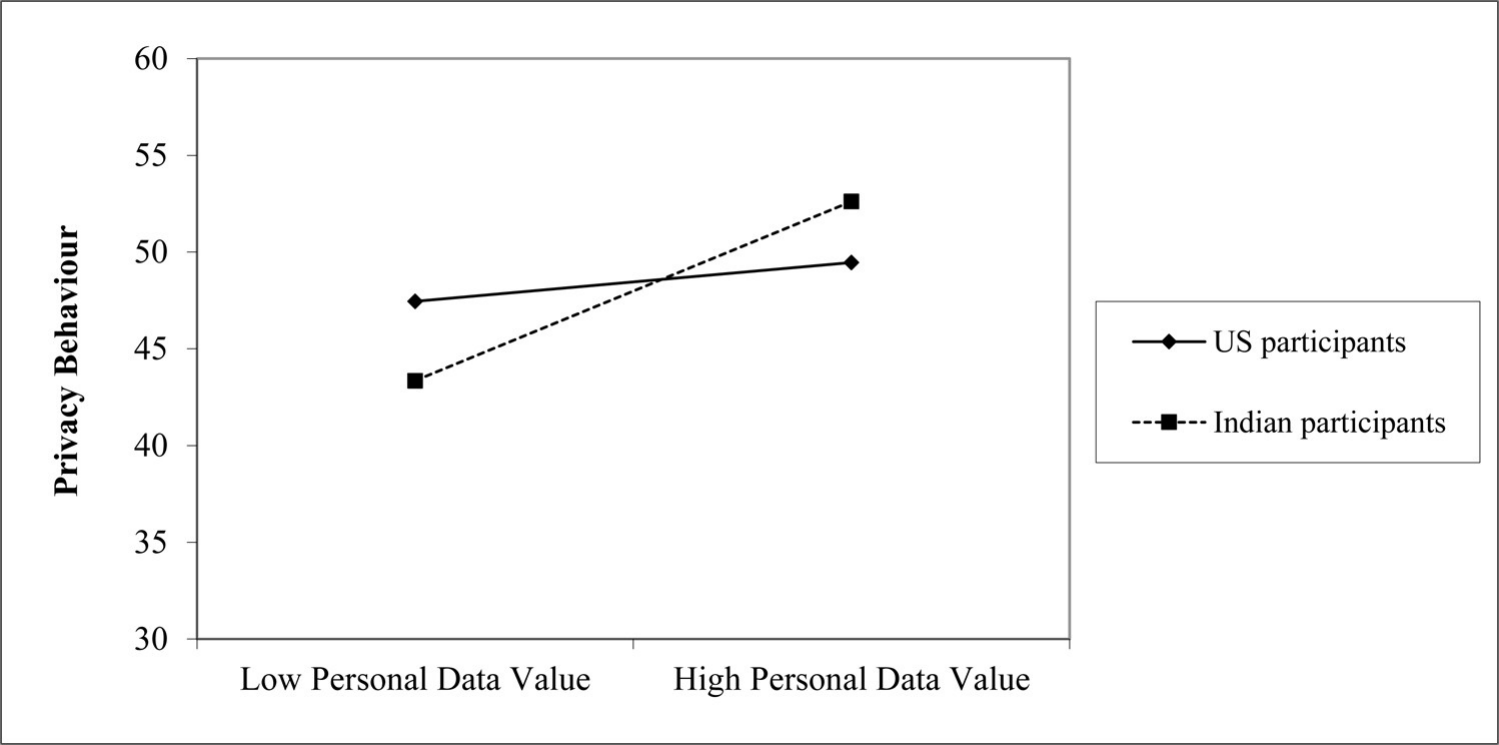
Simple slopes of personal data value by country. Points plotted on the x-axis are one SD above and below the mean.

As in Study 1a multiple regressions were carried out on the US and Indian samples to predict self-reported privacy behaviours based on privacy concern, and value (see Table 4). A dummy variable of country was included. We were specifically interested in the interaction between country and value on privacy behaviour–on the basis that in a collectivist culture, in which personal data is more valued, that personal data value will have a greater impact on behaviour. This interaction term was introduced at a second step. Unlike the UK (Study 1a) data there was a direct prediction of privacy behaviour from personal data value, and, like the UK (Study 1a) data, an effect of privacy concern, but no main effect of country. The interaction term contributed to a significantly better model and revealed the Indian participants’ behaviour was sensitive to personal data value, unlike the US participants (see Fig 1 for simple slopes). Again, the pattern of results is the same if the available scores from attention-filtered participants were also included.

**Table 4:** Regression analysis predicting privacy behaviour from demographic variables, privacy concern, mean-centred personal data value, and country (India or USA), Study 1b.

Model	Variable	B	95% CI		SE	Beta	t	*p*	F	R-squared	R-squared change
1	Constant	26.630	(20.310,	32.949)	3.218		8.275	< .001	18.084	.126***	
	Age	0.054	(-0.030,	0.139)	0.043	.049	1.268	.205			
	Gender (1 = Female)	1.708	(-0.106,	3.523)	0.924	.071	1.849	.065			
	Privacy Concern (CFIP)	0.219	(0.150,	0.288)	0.035	.247	6.255	< .001			
	Personal Data Value	0.090	(0.043,	0.137)	0.024	.169	3.768	< .001			
	**Country (1 = India)**	0.465	(-1.526,	2.455)	1.014	.020	0.458	.647			
2	Constant	27.437	(21.146,	33.728)	3.204		8.565	< .001	17.051	.141***	.014**
	Age	0.057	(-0.027,	0.141)	0.043	.051	1.337	.182			
	Gender (1 = Female)	1.835	(0.032,	3.638)	0.918	.076	1.999	.046			
	Privacy Concern (CFIP)	0.203	(0.134,	0.272)	0.035	.229	5.777	< .001			
	Personal Data Value	0.047	(-0.007,	0.100)	0.027	.087	1.715	.087			
	**Country (1 = India)**	-0.472	(-2.528,	1.583)	1.047	-.021	-0.451	.652			
	**Value X Country**	0.170	(0.067,	0.272)	0.052	.165	3.242	.001			

Note

** *p* < .01

*** *p* < .001.

### Discussion

Privacy concern and personal data value were associated with privacy behaviour; however, the effect of personal data value was primarily driven by the Indian participants. Overall, personal data perceptions were higher than in Study 1a and higher for the Indian participants than the US participants. Although personal data value was associated with privacy concern it shows a distinct pattern of association with behaviour and country of residence. Participant behaviour was better predicted in this study compared to Study 1a.

The overall model is considerably better at explaining behavioural variance for the US and Indian participants than that for the Study 1a, UK sample. The improvement can be attributed to the variance accounted for by the effect of personal data value and specifically the differentiated role it played in US and Indian participants’ privacy behaviour. In this way personal data value may partly explain the relationship between privacy concern and privacy behaviour. Social importance of group relations in India might account for our findings that personal data is highly valued, which could be examined in further research. The direct prediction of behaviour from personal data value suggests the importance of data value in a collectivist society: individual differences in views on personal data value predict privacy behaviour. Therefore, Indian participants, who come from a more collectivist culture, may be more sensitive to their own personal data value–which might be high or low; they were more likely to have privacy behaviours consistent with their perceived data valuations than US participants; this may be due to the social importance of accidental exposure of sensitive personal data in India. There are known cultural differences between the US and India around independence and interdependence. However, there are some limitations of this study. The samples are not exactly equivalent–the Indian sample has fewer females–which can be associated with decreased disclosure (e.g. [6]) and have slower completion times. These may be due to greater engagement or reduced English fluency. Although it was not our goal to generalise these results to national populations, an mTurk sample cannot do so; particularly for one where English is not the primary language. Indeed, there are many potential differences between the US and Indian samples, culturally, contextually and demographically. For example, differences in family income may influence disclosure rates in a similar manner [6]. Therefore, although our theoretical perspective indicates that broad levels of interdependence should relate to the differing effect of personal data value, we cannot say that for certain following this study. Therefore, we examine this in study 2.

One further difference between the samples is that the mTurk-recruited participants in Study 1b had greater technological familiarity. mTurkers are known to require a minimum level of digital literacy both to be aware of crowd work and to use the interface and this was apparent when we compare the 1a and 1b samples [68]. It is usually the case that amongst more experienced/technically proficient groups intention-behaviour links are stronger [69]. The mTurker-recruited sample may also have above average understanding of technology, and by inference, data disclosure and social media for whom personal data value, privacy concern and privacy behaviour are likely to be more meaningful. These combined factors might partly explain the better prediction of privacy behaviour.

## Study 2 –UK crowd-sourced sample

Study 2 bridges the gap between studies 1a and 1b and examines one way in which cultural differences might account for the differential relationship between personal data value and privacy behaviour. Study 1a used UK participants and found personal data value did not predict privacy behaviour. Study 1b used Indian and American participants and found a value-behaviour association for Indian participants. This study uses UK participants but recruited in a similar way to Study 1b, and therefore they are likely to have equivalent technological proficiency to the Indian and American participants. Furthermore, this study examines cultural differences via self-construal at an individual level, rather than at a population level.

Cross-cultural differences can benefit from assessment at a fine-grained individual level instead of using a population level approach. Past research has commonly used broad country-wide categorisations based upon population data. These categorisations show India as more collectivistic than the United States [70]. However, this binary view of Eastern versus Western is very broad and implies the idea that individualism is exclusive of collectivism. However, these broad conceptions do not always predict cross cultural differences [71]. Furthermore, people are not uniform within a country and individual variability should be considered [72]. A recent study proposed seven dimensions of independence-interdependence that can be considered at both individual and cultural levels using measures of self-construal. Two sub-scales, similar-different and harmony-self-expression are of particular interest as they are profoundly different between Western and Southern/Eastern Asian populations [73]; Study 1b explored the difference between US and Indian participants and therefore subscales which differentiate between these two groups will be the focus of this study. Both the similar-different and self-expression-harmony subscales were significantly higher than average for the Western samples (more different and self-expressive) and significantly lower than average for the Southern/Easter Asian populations (more similar and harmonious); they are therefore the best candidates to explain differences from study 1b. The similar-different subscale refers to a way of defining the self as either unique or as ‘fitting in’; the harmony-self-expression subscale refers to communicating with others either forthrightly or to avoid upsetting others. These fine-level cross-cultural differences may well account for the difference between US and Indian participants association of personal data value with privacy behaviour. We predict that characteristically collectivist self-construals, similar and harmonious, will increase the effect of privacy value on privacy behaviour in the same way that Indian nationality did in Study 1b.

Study 2 also addresses some potential confounds from Study 1b. Participant recruitment is again crowd-sourced however a UK provider instead of mTurk and British participants are used to allow comparability to Study 1a. Differences between groups in Study 1b could be attributed to non-psychological differences between the US and India including different laws, environment or wealth. By looking at individual differences within a single country these issues are addressed and self-construal differences which may underpin individualist-collectivist differences in perceived privacy concern, value and behaviour can be examined.

### Method

#### Participants

This study was also approved by UEA School of Psychology ethics committee: 2016-0040-000316. Consent was obtained online at the commencement of the study. A sample of UK nationals were invited to take part in the study via Prolific Academic. The final sample consisted of 442 participants; 517 began the survey, 36 withdrew/failed to complete the survey, 38 participants were removed for failing attention filters and 1 was removed for a total completion time under five minutes (under 1/3 median response time). The final sample was 67.9% female with an average age of 36.3 years (*SD* = 11.18). Median completion time was 17.5 minutes.

#### Materials and procedure

The procedure was very similar to Study 1a with the addition of a seven-dimensional 22-item self-construal scale [73] between the concern for information privacy scale and the privacy behaviour scale. The self-construal scale was answered on a 9-point Likert-type sale for how closely the statements described the respondent from 1 *not at all* to 9 *exactly*. Two sub-scales were of interest. Firstly, Similar-Different with four items, two reverse-scored, e.g. *You like being different from others; Being different from others makes you feel uncomfortable (reversed);* higher scores indicate greater self-definition as unique (α = .699). Secondly, Harmony-Self-Expression with three items, one-reverse scored, e.g. *You show your inner feelings even if it disturbs the harmony in your family;* higher scores indicate greater forthright self-expression (α = .413). Given the low reliability for Harmony-Self-Expression the reverse-scored item was removed to produce a 2-item scale (α = .602). Survey scale reliabilities remained high for the CFIP (α = .891) and the privacy behaviour scale (α = .821), see also Table 1.

### Results

Personal data perceptions across the five dimensions (perceived data value, ownership, legal ownership, control and security) were significantly higher than the Study 1a UK sample (*t* > 4.5, *p* < .001) and fell between the Indian and US samples from Study 1b (*t* > 2.4, *p* < .015), see Table 1. Personal data value in particular was about 9% above the UK Study 1a valuations but about 22% lower than the Indian participant valuations and about 5% above the US participant valuations. Variance in the latest sample was roughly equivalent to the US sample. Furthermore, as expected technological familiarity in this crowd-sourced UK study (*M* = 54, *SD* = 12) was significantly higher than the first UK study sample (*M* = 44, *SD* = 16, *p* < .001); the Indian mTurk sample was more technologically familiar than the current sample (*M* = 61, *SD* = 9, *p* < .001) and the US was not significantly different by Bonferroni corrected comparison (*p* = .504).

Preliminary correlations revealed a pattern part-way between Study 1a and 1b with significant correlations between personal data value, privacy concern and privacy behaviour but smaller than those found in Study 1b, see Table 5. The self-construed Difference-Similarity and Harmony-Self-Expression were significantly associated with each other and with privacy behaviour but not with personal data value. Self-construed Difference-Similarity was associated with privacy concern.

**Table 5:** Correlation coefficients for research variables in Study 2.

	Personal Data Value		Privacy Concern (CFIP)		Privacy Behaviour		SC—Difference		SC–Self-Expression	
Personal Data Value										
Privacy Concern (CFIP)	.153	**								
Privacy Behaviour	.096	*	.280	***						
SC—Difference	-.044		.145	**	.197	***				
SC–Self-Expression	-.043		.055		.170	***	.241	***		

Note

* *p* < .05

** *p* < .01

*** *p* < .001.

As in studies 1a and 1b multiple regressions were carried out to predict self-reported privacy behaviours based on privacy concern, and personal data value (see Table 6) as well as self-construed Difference-Similarity and self-construed Harmony-Self-Expression. We were specifically interested in the interaction between the self-construals and personal data value on privacy behaviour–on the basis that a desire to be more similar/harmonious with others would mirror the collectivist cultural effect from Study 1b with a greater impact for value on behaviour for those individuals. An initial model matched the variables from Study 1a, the self-construal variables were added at a second step and the interaction terms were introduced at a third step. The interaction of Harmony-Self-Expression with personal data value did not reach significance and so was omitted from the final model. Inclusion/omission of that non-significant interaction term does not change the pattern of results reported here. As in Study 1a and Study 1b there was a main effect of Privacy Concern. Self-construals as different and self-expressing were associated with increased privacy behaviour. As in Study 1a there was no effect of personal data value–until the interaction of Difference-Similarity and personal data value was introduced at Step 3.

**Table 6:** Regression analysis predicting privacy behaviour from demographic variables, privacy concern, personal data value, self-construal difference, and self-construal self-expression.

Model	Variable	B	95% CI		SE	Beta	t	*p*	F	R-squared	R-squared change
1	Constant	17.274	(8.573,	25.975)	4.427		3.902	< .001	12.192	.100	
	Age	0.121	(0.038,	0.204)	0.042	0.132	2.873	.004			
	Gender (1 = Female)	-0.678	(-2.641,	1.286)	0.999	-0.031	0.678	.498			
	Privacy Concern (CFIP)	0.258	(0.166,	0.35)	0.047	0.255	5.499	< .001			
	Personal Data Value	0.026	(-0.02,	0.072)	0.023	0.051	1.105	.270			
2	Constant	19.530	(10.925,	28.134)	4.378		4.461	< .001	12.104	.143***	.043***
	Age	0.129	(0.047,	0.21)	0.041	0.141	3.107	.002			
	Gender (1 = Female)	-0.404	(-2.329,	1.521)	0.979	-0.018	-0.413	.680			
	Privacy Concern (CFIP)	0.228	(0.137,	0.32)	0.046	0.225	4.911	< .001			
	Personal Data Value	0.033	(-0.012,	0.078)	0.023	0.066	1.459	.145			
	**SC—Difference**	0.211	(0.057,	0.364)	0.078	0.125	2.696	.007			
	**SC—Self-Expression**	0.383	(0.136,	0.629)	0.125	0.14	3.053	.002			
3	Constant	19.560	(11.001,	28.118)	4.354		4.492	< .001	11.301	.154***	.011*
	Age	0.133	(0.052,	0.214)	0.041	0.146	3.224	.001			
	Gender (1 = Female)	-0.276	(-2.193,	1.642)	0.975	-0.013	-0.282	.778			
	Privacy Concern (CFIP)	0.225	(0.134,	0.316)	0.046	0.222	4.859	< .001			
	Personal Data Value	0.047	(0.001,	0.093)	0.023	0.093	2.014	.045			
	**SC—Difference**	0.188	(0.034,	0.342)	0.078	0.111	2.394	.017			
	**SC—Self-Expression**	0.395	(0.15,	0.64)	0.125	0.145	3.166	.002			
	**Value X SC Difference**	-0.009	(-0.016,	-0.002)	0.004	-0.11	-2.387	.017			

Note—The latter three variables were mean-centered.

* *p* < .05

*** *p* < .001.

**Fig 2:**
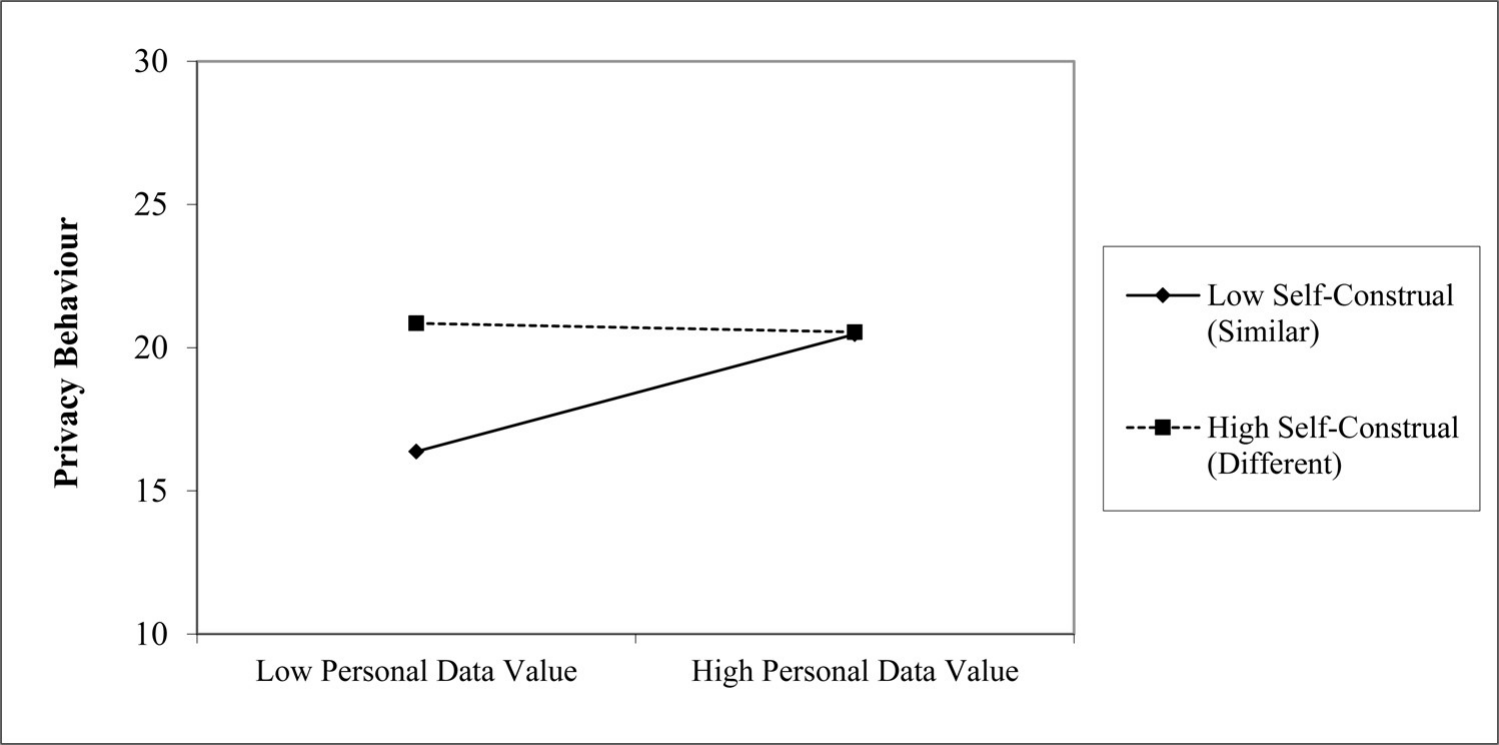
Simple slope of value by self construal. Points plotted on the x-axis are one SD above and below the mean.

The interaction result shows participants with a desire for low difference/high similarity to others were highly sensitive to personal data value, but this effect decreased with increasing preference for difference (see Fig 2). The interaction term contributed to a significantly better model. The results retain the same pattern if excluded participants (attention filter and fast completion time) are included.

### Discussion

Privacy concern and personal data value were associated with privacy behaviour; however, the effect of privacy data value was primarily driven by participants who had self-construals as similar–to-others. Personal data perceptions were higher than the Study 1a UK participants and the Study 1b US participants but lower than the Indian participants from Study 1b.

The overall model is slightly better than the regression for Study 1b and considerably better than Study 1a. This may be partly due to the relatively greater technological familiarity which was similar to the US participants in Study 1b. It may also be partly due to the increased sensitivity of including individual differences in self-construal instead of country-level cultural differences.

Although this is an exploratory study, one account is that self-construed similarity-difference to others might explain the cultural difference in Study 1b. Existing evidence shows greater self-construed similarity in Southern/Eastern Asian populations such as India and greater self-construed difference in Western populations such as the US [73]. Self-construed similarity and Indian residency both demonstrated increased sensitivity to personal data value, whereas self-construed difference and US residence demonstrated almost no sensitivity to personal data value. An associated psychological account would be that culturally in India there is a greater emphasis on belonging to a group as illustrated by self-construal of similarity. For those who wish to belong to a group privacy behaviour becomes more important for valued data–which is characterised by ownership, security and control. This account would imply a greater emphasis on keeping data secure which is currently private, but less effort to secure data which has already been shared. This account is consistent with group cohesiveness being maintained by sharing low-value information and securing high-value information which might lead to conflict. This issue would not be important for those who self-construe as different as there is no need to maintain a profile of similarity with others (consistent with evidence elsewhere [6]). Perhaps surprisingly, self-construal as non-harmonious/self-expressing did predict greater privacy behaviour but did not moderate personal data value. Relatedly, self-construal as *different from others* was associated with greater privacy behaviour on average. However, these results can be explained as a result of a desire to share low-value information for those who wish to enhance their group belonging–and therefore relatively less privacy behaviour overall from self-construal as *similar to others* and *harmonious with others*.

There are limitations to this study and alternative accounts are possible. The study sample is not representative of the UK population and may not generalise beyond the sampled population. Given the cultural nuances, values and socio-demographics that might influence privacy it may be that a variable associated with both privacy behaviour and the measured self-construals underlies the measured effect. Further research to confirm the finding and measure a wider range of socio-demographic and socio-cultural individual differences compared across specific contexts would be a next step to exploring the results found here.

Overall, the effect of self-construed similarity-difference provides one account in which personal data value becomes important to privacy behaviour. This could explain the failure to find an effect of personal data value in Study 1a and can explain the cultural differences in Study 1b as a consequence of participants’ approach to interpersonal relationships.

## General discussion

These studies have shown that personal data value can be meaningful in predicting privacy behaviour in addition to privacy concern. Although it is difficult to value personal data with any precision, and although Study 1a showed no effect of value, Studies 1b and 2 demonstrate that there are differences in personal data value cross-culturally and that combined with individual differences they can predict privacy behaviour. More specifically, self-construal as similar and/or Indian residency increased sensitivity to personal data value as a predictor of privacy behaviour. This moderating factor offers an explanation as to why evidence to date has been mixed in assessing the importance of personal data value.

This research provides a potential avenue to explain why some people are consistently more willing to sell personal data than others. The studies reported here support the increasingly corroborated view that privacy management is influenced by multiple aspects as suggested by ‘privacy calculus’ [12, 13]. Furthermore, we would suggest that personal data value is an important factor, distinct from privacy concern and privacy risk which is weighed more heavily in privacy management decisions amongst individuals who self-construe as similar to others.

Although privacy management is partly defined by context our data suggests a further potential mechanism to target an intervention in order to reduce the mismatch between people’s privacy behaviour and stated concern [7]. Here we show that people report behaving according to their concerns about their data to a greater extent if they value that data more. Therefore, any intervention that can increase the extent to which an individual values their data may prove fruitful in predicting and encouraging appropriate privacy management. Further, our data show that interventions designed to increase social cohesion, such as highlighting social identities [74]; may also increase the perceived value of social data. Similarly, a social norms approach showing that others belonging to meaningful groups value their personal data is also likely to promote positive behaviour [75].

This finding is consistent with work showing socio-cultural effects of self-disclosure within the US population [6]. One finding has been that socio-demographics account for considerable variability in self-reported political self-disclosure on social media; this includes findings that individuals who are older, female and/or less educated were less likely to self-disclose. A further finding was that ethnicity moderated the interactions social media connections, self-disclosure and social media use. Our study suggests that self-construal and its impact on privacy sensitivity might partly explain the role of ethnicity in this interaction.

The motivations that underlie online privacy behaviour are complex and likely include characteristics unique to individual documents, websites or apps [7, 12, 76]. One tangible and important example is the sharing of medical data, which is becoming ubiquitous, but has yet to develop a ‘norm’ [77]. Park and Shin [78] found the privacy paradox to be at play in this domain, while highlighting the complexities of variables influencing both privacy attitudes and behaviours. Importantly Park and Shin show that attitudes can predict behaviour indirectly, via the want to share–some people might very strongly wish to share their data, knowing the wide benefits this might have in a medical context. Thus, future work, and in particular work interested in medical data sharing, may benefit from considering data-value as we highlight here and how this influences data sharing, especially when ‘value’ may also become contextual as the data might have value to help others and not only help or harm oneself.

Future research would do well to examine the importance of self-construal for different types of personal data. Information regarding socially unacceptable behaviour is known to be more valuable in general [23], likely more so for people who self-construe as similar. For a more complete picture, other individual difference variables should be examined in relation to privacy attitudes and behaviour; there is evidence that greater agreeableness and conscientiousness are associated with willingness to waive privacy, and that greater extraversion and agreeableness are associated with positive views of technology that requires personal information [76, 79]. Further, as the studies presented here are correlational, it is crucial that future work aims to experimentally manipulate some of the variables that the current work highlights as important to information privacy. For example, future work might look experimentally at the role of value and self-construal. It will similarly be important for future work to assess whether there are any yet-to-be-examined variables that contribute to our observed cross-cultural differences. Finally, data perceptions including perceived control, security, personal and legal ownership were highly intercorrelated to the point where we cannot meaningfully distinguish these constructs. However, these constructs differ in theory, and perhaps there are situations in which these variables can be disassociated. For example, one may strongly feel ownership for a selfie posted on Reddit, but unable to secure it, if it becomes popular and widely shared.

The mixed evidence regarding the privacy behaviour—concern relationship is partly explained by the difficulty in assessing the value of personal data, which might inform our concern and guide our behaviour. However, there is evidence for a meaningful role for value, which may have previously been overlooked due to the moderating effect of individual differences. This research has identified self-construal as similar to others as a factor in increasing sensitivity to value. Privacy concern, privacy value and self-construed social beliefs all have a role in privacy behaviour. This partly explains incautious privacy behaviour for information that is self-perceived as having low personal value but would be potentially harmful in the wrong hands.

## Supporting information

S1 Dataset Study 1a dataset, SPSS format. (SAV)

S2 Dataset Study 1a SPSS syntax. (SPS)

S3 Dataset Study 1b dataset, SPSS format. (SAV)

S4 Dataset Study 1b SPSS syntax. (SPS)

S5 Dataset Study 2 dataset, SPSS format. (SAV)

S6 Dataset Study 2 SPSS syntax. (SPS)

S1 File Personal data perception questions. (DOCX)
