# Supplementary material for: The role of personal data value, culture and self-construal in online privacy behaviour
Source: PLoS One. 2021 Jul 16;16(7):e0253568. doi: 10.1371/journal.pone.0253568 (PMC8284788; doi:10.1371/journal.pone.0253568)
Supplement: S1 File — (DOCX) [file pone.0253568.s007.docx]

**S1 File. Personal Data Perception Questions**

Our location can now be tracked (GPS coordinates) via many devices. [For the below, imagine that they refer to your location throughout the day /For the below, imagine that you have location data for yourself, throughout each day, and have stored it on your own cloud storage (online, e.g. Dropbox, One Drive, iCloud, Microsoft cloud).].

Please indicate to what extent you agree with the following statements.

I feel like I own a week’s GPS coordinates of myself, [stored on a personal device (e.g. a running watch) / stored on a cloud / while at home /while at work / for the last week /for a week, from 3 years ago].

I am in control of a week’s GPS coordinates of myself, [stored on a personal device (e.g. a running watch) / stored on a cloud / while at home /while at work / for the last week /for a week, from 3 years ago].

I legally own a week’s GPS coordinates of myself, [stored on a personal device (e.g. a running watch) / stored on a cloud / while at home /while at work / for the last week /for a week, from 3 years ago].

A week’s GPS coordinates of myself, [stored on a personal device (e.g. a running watch) / stored on a cloud / while at home /while at work / for the last week /for a week, from 3 years ago] are valuable to me.

A week’s GPS coordinates of myself, [stored on a personal device (e.g. a running watch) / stored on a cloud / while at home /while at work / for the last week /for a week, from 3 years ago] are relatively secure.

For the below, please assume that the unsent text message is saved as a draft on your handset. / For the below, please assume that you sent the text message from your phone. / For the below, please assume this refers to a message you have sent to another person via WhatsApp (a smartphone app for instant messaging). / For the below, please assume that this refers to a Tweet (a 140 character-limited statement made on Twitter.com) you have published in an unrestricted fashion, so that anyone can see it. / For the below, please assume these are text message that you have sent (via your network provider) from your handset.

I feel like I own [an unsent text message / a sent text message / a WhatsApp message/ a Tweet (from a non-restricted profile)] / the text messages I have sent during the last week / the text messages I have sent during one week, from 3 years ago].

I am in control of [an unsent text message / a sent text message / a WhatsApp message/ a Tweet (from a non-restricted profile)] / the text messages I have sent during the last week / the text messages I have sent during one week, from 3 years ago].

I legally own [an unsent text message / a sent text message / a WhatsApp message/ a Tweet (from a non-restricted profile)] / the text messages I have sent during the last week / the text messages I have sent during one week, from 3 years ago].

[An unsent text message / A sent text message / A WhatsApp message/ A Tweet (from a non-restricted profile)] / The text messages I have sent during the last week / The text messages I have sent during one week, from 3 years ago] is/are valuable to me.

[An unsent text message / A sent text message / A WhatsApp message/ A Tweet (from a non-restricted profile)] / The text messages I have sent during the last week / The text messages I have sent during one week, from 3 years ago] is/are relatively secure.

For the below, please assume that this is a picture that you took of yourself (a selfie), [and have stored on your own cloud storage (online, e.g. Dropbox, One Drive, iCloud, Microsoft cloud) /that you have stored on your own portable storage device (e.g. a portable hard drive, USB, memory card) / and have shared on your own account / and have stored it only on your handset ].

I feel like I own a selfie [on a portable storage device /on cloud storage / I have posted on Facebook / on my phone / I have taken in the last week / I have taken 3 years ago].

I am in control of a selfie [on a portable storage device /on cloud storage / I have posted on Facebook / on my phone / I have taken in the last week / I have taken 3 years ago].

I legally own a selfie [on a portable storage device /on cloud storage / I have posted on Facebook / on my phone / I have taken in the last week / I have taken 3 years ago].

A selfie [on a portable storage device /on cloud storage / I have posted on Facebook / on my phone / I have taken in the last week / I have taken 3 years ago] is valuable to me.

A selfie [on a portable storage device /on cloud storage / I have posted on Facebook / on my phone / I have taken in the last week / I have taken 3 years ago]is relatively secure.
